# Unconventional Strategies for Aphid Management in Sorghum

**DOI:** 10.3390/insects15070475

**Published:** 2024-06-26

**Authors:** Ivan Grijalva, Qing Kang, Daniel Flippo, Ajay Sharda, Brian McCornack

**Affiliations:** 1 Department of Entomology, Kansas State University, Manhattan, KS 66506, USA; mccornac@ksu.edu; 2 Department of Statistics, Kansas State University, Manhattan, KS 66506, USA; qkang@ksu.edu; 3 Department of Biological and Agricultural Engineering, Kansas State University, Manhattan, KS 66506, USA; dkflippo@ksu.edu (D.F.); asharda@ksu.edu (A.S.)

**Keywords:** field crops, sorghum, integrated pest management, insecticides, sorghum aphid

## Abstract

**Simple Summary:**

Sorghum holds significant economic importance across both the northern and southern regions of the U.S. Since 2013, the presence of the sorghum aphid *Melanaphis sorghi* (Theobald) has led to -yield reduction in unmanaged sorghum. Various management strategies have been critical in controlling aphid infestations, including cultural practices, host–plant resistance, and insecticide use. Typically, the common practice is applying insecticides at a whole-field level once aphids reach they reach a certain economic threshold. However, this traditional approach often includes treating plants that are not yet infested. To minimize insecticide usage in sorghum, we suggested selectively spraying individual plants only when aphids are present or not, as opposed to conventional spraying based on economic thresholds. Through field experiments conducted over two summer seasons, our findings demonstrated that spraying individual plants can manage aphid populations while reducing overall insecticide usage. This approach holds promise for advancing targeted insecticide applications in sorghum.

**Abstract:**

Since the invasion of the sorghum aphid *Melanaphis sorghi* (Theobald), farmers in the sorghum (*Sorghum bicolor* L. Moench) production region in the Great Plains of the U.S. have faced significant crop damage and reduced yields. One widely used practice to aid in managing sorghum aphids is pest monitoring, which often results in field-level insecticide applications when an economic threshold is reached. However, relying on this traditional management practice includes the application of insecticides to non-infested plants. To reduce insecticide usage in sorghum, we proposed spraying individual plants when aphids are present or absent compared to traditional spraying based on a standard economic threshold using field replicate plots over two summer seasons. The experimental results of this study indicated fewer aphids in plots managed with an economic threshold, followed by randomly sprayed and plant-specific treatments compared with the untreated control treatment. Therefore, compared with traditional management, those treatments can be alternative strategies for managing aphids on sorghum within our field plot study.

## 1. Introduction

Agricultural pests represent 37% of annual crop losses, 13% of which are directly caused by insects [1]. Advantageously integrated pest management was promoted in the early 1970s as a strategy for pest control that promotes sustainable agriculture with a strong ecological basis [2]. Different strategies have been used in integrated pest management to manage insect pests in agriculture, including the application of insecticides, cultural practices, host–plant resistance, and mechanical and biological management methods [3]. One of the most widely used strategies for managing insect pests is the use of insecticides; however, there is much concern about their use for pest control due to their cumulative adverse effects on the environment [4,5,6].

Primary concerns include the effects of insecticides on non-target or beneficial organisms, including natural enemies and pollinators [7,8], and the potential for developing insecticide resistance [9,10]. Additionally, the use of insecticides can create indirect costs, including negative impacts on the human health of growers or consumers (e.g., poisoning), as well as long-term effects on the environment (e.g., bioaccumulation, biodiversity, etc.) [11,12]. Therefore, given the various negative impacts of insecticides and the reliance on single strategies to manage key pests, there is a need to find alternatives to reduce the use of insecticides to promote sustainability in field crops [4].

Sorghum (*Sorghum bicolor* (L.) Moench) is a field crop that relies on insecticide application to manage pests. In recent years, fields infested by sorghum aphid (*Melanaphis sorghi* (Theobald)) [13] have significantly impacted production in the Southern Great Plains, which has increased the use of seed-applied and foliar insecticides [14]. Kansas is one of the major sorghum-producing states in this region, and sorghum aphid infestations have reduced yields since 2015 [15]. The use of resistant hybrids and the management of aphid populations early in the southern range of its distribution have reduced the overall impact of this pest on sorghum. However, sorghum aphids reach damaging levels in sorghum production areas across the state.

Damage caused by sorghum aphids includes leaf chlorosis, a reduction in plant nutrients, and decreased photosynthesis due to honeydew secretion and sooty mold growth [16,17]. During pre-flowering, aphid infestations can decrease the number of heads, seed weights, and plant maturity, causing a reduction in yield [15]. Due to their high reproductive rates and ability to disperse over great distances, usually during their alate stage with the wind, monitoring changes in aphid populations is imperative for the timely and warranted use of insecticides, which are applied based on established economic thresholds [17].

Traditional aphid management, which depends on published economic thresholds, suggests the application of insecticides when 20–30% of sorghum plants are infested with an estimated 25–125 sorghum aphids/wingless/leaf threshold [18]. Others suggest the use of a 40 sorghum aphid wingless/leaf threshold or “tally threshold” for insecticide treatment [19]. Usually, thresholds and related sampling plans rely on mathematical probabilities that unsampled plants will require treatment based on aphid densities using subsamples of the entire plant population. For example, areas or unsampled plants with low or no aphid presence can be sprayed unnecessarily, resulting in a needless increase in the use of insecticides. Whole-field insecticide applications require high amounts of insecticides to treat a crop’s area, resulting in significant production costs and an increased environmental impact [20]. Additionally, spraying an entire field with insecticides could increase the risk of aphid resistance [14] and reduce the number of predators and parasitoids contributing to natural predation by sorghum aphid populations.

Therefore, to reduce the use of insecticides on unnecessary areas or non-infested plants with aphids, we propose a field plot study in which individual plants are sprayed with or without aphids compared to traditional spraying based on standard economic thresholds. Sorghum was used as a crop model to evaluate the effect of plant-level applications on sorghum aphid populations and yield differences. We suggest that our approach could be used to manage sorghum aphid populations while reducing insecticide use by monitoring aphid pests and only applying insecticides at the plant level when aphids are present or absent.

## 2. Materials and Methods

### 2.1. Site Location

This research study was conducted in fields located in Manhattan, KS, with coordinates of (39°07′33.6″ N, 96°38′09.6″ W) and (39°12′50.4″ N, 96°35′49.2″ W). The fields were planted with 0.20 ha of sorghum using susceptible seeds of varieties 84P80 and 84G62 (Pioneer, Johnston, IA, USA) at a seeding rate of 60,000 seeds/acre in 2021 and 2022, respectively. The aphid tolerance of the seeds was rated as 2 out of 10, which is considered “poor” according to the company. The sorghum plants were spaced at 0.76 m between rows using no-till practices and were planted in June during the 2021 and 2022 growing seasons. Several herbicides were sprayed to manage weeds using a boom sprayer, including Brawl II (2046 mL/ha) (Tenkoz, Alpharetta, GA, USA), Atrazine 4L (3069.27 mL/ha) (Syngenta, Greensboro, NC, USA), Explorer (394.62 mL/ha) (Syngenta, Greensboro, NC, USA), and Warrant (5846.22 mL/ha) (Bayer CropScience, St. Louis, MO, USA). No fertilizers were applied during this study, and insecticide was applied to manage sorghum aphid populations.

### 2.2. Insecticide Applications

The insecticide Sivanto^TM^ prime (Flupyradifurone) (Bayer CropScience, St Louis, MO, USA) was applied at a standard commercial rate (4 fl oz/acre) of 292.31 mL/ha for 4 treatments (Table 1). This was completed using a backpack pump sprayer with dimensions of 22.86 cm × 39.37 cm × 52.07 cm that was equipped with a Teejet 110015 nozzle. The untreated control plots did not receive insecticide during the duration of this study. The economic threshold plots were sprayed with a broad-spectrum insecticide on all plants in the two middle rows when they reached an economic threshold of ≥40 mean wingless aphids per leaf on 12 evaluated plants. In contrast, the randomly sprayed and plant-specific plots were treated based on the presence or absence of aphids on individual plants, simulating the actions of a ground robotic vehicle. Finally, the tally threshold plots were sprayed based on an economic threshold of ≥40 wingless sorghum aphids per plant on 12 evaluated plants.

Various spray applications on individual plants were conducted at different phases for the randomly sprayed, plant-specific, and tally threshold treatments. However, in 2021 and 2022, individual plots were sprayed only once at different phases because the plot replicates did not simultaneously reach the economic threshold, due to typical variations in aphid populations. The insecticide was applied on a weekly basis according to the treatments outlined in Table 1.

### 2.3. Field Design and Treatments

In this study, we used a randomized complete block design to assess the effects of individual plant insecticide applications on the overall dynamics of aphid populations within plots, compared to standard whole-plot insecticide applications based on established economic thresholds. The sorghum fields were divided into 30 plots. Each plot was 4 rows wide by 12 m long, and each block was spaced 3 m apart from the other. The field experiments in both years consisted of 5 randomized treatments within each of the 6 blocks.

The location of the plots in the field served as the blocking factor. The 5 treatments were randomly assigned to a plot within each block. Each treatment was represented by six plots. The counts of wingless aphids were visually assessed at 15 sampling days, including 3 days at baseline, 1 day (1D), and 6 days (6D) after insecticide applications, and they were grouped into evaluation phases or weeks (6 in total) to observe the efficacy of the treatment over time. The goal was to see if randomly sprayed, and plant-specific are feasible treatments compared with the economic threshold, as these treatments simulate the feasibility of deploying a ground robotic vehicle capable of detecting or not aphids and spraying individual plants.

Sorghum aphids were counted from 2 leaves of 12 randomly selected plants on each sampling day, with different plants being selected randomly for each sampling day. This same experimental design was used for the 2021 and 2022 summer seasons.

### 2.4. Non-Natural Infestations of Sorghum Aphid Populations

**Figure 1:**
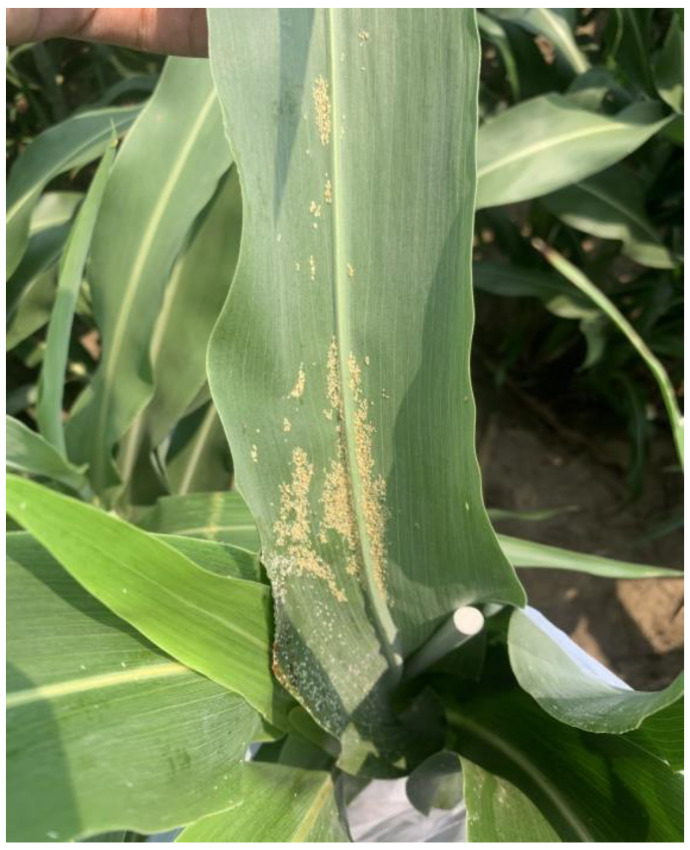
Sorghum plants manually infested with wingless aphids.

**Figure 2:**
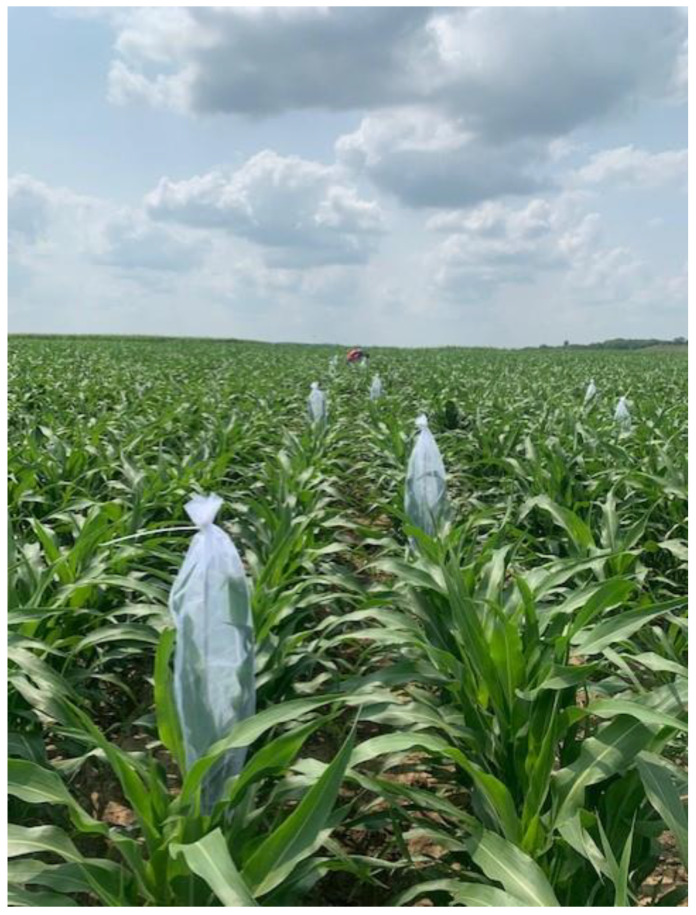
Sorghum plants infested with wingless aphids were covered with a sleeve cage.

Based on preliminary field experiments conducted in the summer of 2020, various sorghum plots did not reach sorghum aphid economic threshold levels for insecticide applications. For this reason, we manually infested 8 random, individual plants with approximately 200 wingless aphids in the two middle rows of each individual plots when the sorghum plants had 7–10 leaves (stage 3) during the growing seasons of 2021 and 2022, respectively (Figure 1). Sorghum aphids were obtained from a research colony maintained at the Entomology Department of Kansas State University, which was established in 2020 from field-collected aphid populations. After randomly selected sorghum plants were infested, each plant was covered with sleeve cages. Each cage was 50 cm long by 20 cm wide with two openings at the ends built with noseeum mesh long enough to allow for plant growth development while protecting the aphids from natural enemies, especially coccinellids (Figure 2). All sleeve cages were removed two weeks before visual assessments started, which allowed sorghum aphids to spread through the center two rows of each replicate plot.

### 2.5. Insect Sampling

Two months after sorghum was planted (stage 4: the flag leaf was visible [15]), visual counts were made of all wingless aphids, which is the visually observed life stage used for management decisions. Density estimates were performed by randomly selecting 12 plants within the two middle rows of each plot. From each plant, we visually assessed two leaves, the most recent completely unfurled leaf below the flag leaf and the first healthy leaf (>80% of the leaf was green) from the bottom of the plant (designated “top” and “bottom” leaves hereafter) [19,21]. For each leaf, the number of wingless aphids was estimated using standard guidelines that had 7 density categories [22]. The categories included 0 wingless/leaf, 1–25 wingless/leaf, 26–50 wingless/leaf, 51–100 wingless/leaf, 101–500 wingless/leaf, 501–1000 wingless/leaf, and >1000 wingless/leaf. All these visual assessments were performed weekly, after treatment applications and prior to sorghum harvest. Manually infested plants were not subjected to visual assessments but served to increase aphid numbers across all plots to promote aphid establishment.

### 2.6. Cumulative Aphid Days and Sprayed Plants

We calculated the cumulative aphid days (CADs) per plot for the wingless using the procedure described by Lytle et al. [23]. CADs are used to calculate the area under the aphid population curve, and it provides a single number describing the aphid density or “pressure” over the entire growing season [23]. We also recorded the total number of sprayed plants in each plot under the ET, RS, PS, and TT treatments for every insecticide application. The number of sprayed plants was used to calculate the number of cumulative sprayed plants (CSP) in the overall evaluation phases.

### 2.7. Yield Sampling

At sorghum maturity, one middle row was randomly selected in each plot, and sorghum heads from 3 m sections of an inner row were harvested by hand and dried using rackets located in a dark room at 25 °C. We excluded the manually infested plants from all plots during the harvest process. A thresher (Almaco, SSBT-C2, Nevada, IA, USA) was used to remove the seeds from the panicles. The seeds were weighed in grams using an electronic balance (Scout^TM^ Pro, SP202, Parsippany, NJ, USA), and the % moisture content was measured using a grain moisture tester (Agratronix, MT-16, Streetsboro, OH, USA) from each sample per plot.

### 2.8. Data Processing

The number of wingless was averaged over 24 leaves measured in each plot. Accordingly, the lower limit of detection for a plot is 1/24 ≅ 0.042. If a plot had no wingless aphids observed, its count was replaced by half of the detection limit, after which the plot underwent natural log transformation for statistical analysis. 

### 2.9. Statistical Analysis

The natural log of counts post-treatment for each response variable was analyzed using the linear mixed model. The fixed effects of the model included block, treatment (Trt), evaluation phase (EP), sample day (D), and all two-way and three-way interactions between treatment, phase, and sample day (Table 2). The random effect of the model was plotted (an error term vector corresponding to repeated measurements over time). The natural log of the baseline counts one day prior to the 1st evaluation phase served as a covariate. The variance–covariance structure of the plot was taken as either unstructured, first-order autoregressive with heterogeneous variance, or compound symmetry depending on model convergence and fitness criteria. Model fitness was supported by the attributes of conditional studentized residuals. Treatment effects were assessed via back-transformed least squares means (LSMs), their standard errors (SEs), and mean differences. Disregarding the results of the test for three-way interactions, treatment groups were compared at each evaluation phase and sample day. Statistical analysis was performed via Statistical Analysis Software (SAS version 9.4; Cary, NC, USA) PROC MIXED with the option DDFM = KR in the MODEL statement.

The yield and CAD variables were analyzed under the linear model, with fixed effects being block and treatment. Treatment effects were assessed via LSMs, SEs, and mean differences. Statistical analysis was performed via SAS PROC MIXED. All tests were conducted at the 0.05 significance level. Pairwise comparisons were carried out using two-sided tests. No multiplicity adjustment was applied.

## 3. Results

In the following sections, treatment abbreviations consist of untreated control (UC), economic threshold (ET), randomly sprayed (RS), plant specific (PS), and tally threshold (TT). Furthermore, the details of each treatment are outlined in Table 1. The *p* represents the *p*-value for the overall effect of treatment. 

### 3.1. Aphid Abundance over Time in the 2021 Season

Initially, all treatments showed similar results one day after application in the first evaluation phase (*p* = 0.571). However, after six days, treatment differences occurred (*p* = 0.003). TT exhibited higher aphid counts 178 ± 32 (LSM ± SE), while RS had lower counts compared to the others. UC differed from ET, PS, and TT but was similar to RS during the initial evaluation.

**Figure 3:**
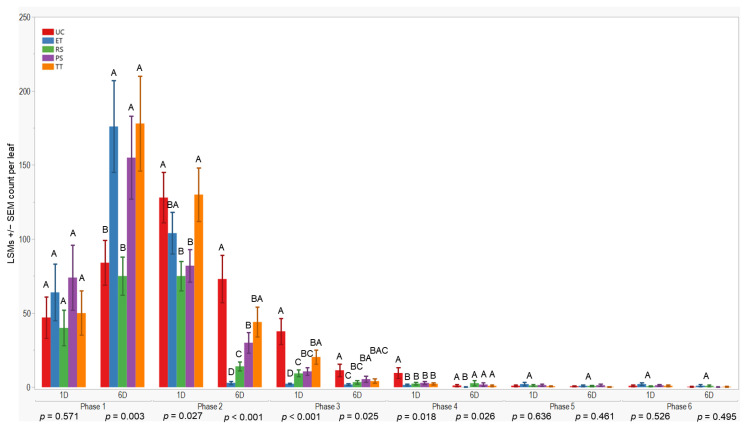
LSMs with SE counts per leaf of wingless in the 2021 season. LSMs with the same letter are not significantly different from each other (*p* >0.05).

In the subsequent evaluation phase, variations were observed one and six days after application (*p* = 0.027; <0.001). RS recorded lower counts one day after application, whereas ET had lower counts six days post-application. Conversely, TT showed higher counts (130 ± 18) one day after application, and UC (73 ± 16) had higher counts six days post-application. Significant differences were observed one day and six days after application in the third (*p* < 0.001; 0.025) and fourth evaluations (*p* = 0.018; 0.026), with UC exhibiting higher counts (≤37.7 ± 8.8) and ET lower counts, respectively. However, no significant differences were observed during the fifth and sixth evaluation phases. In summary, plots treated with ET, RS, and PS consistently exhibited lower aphid counts (≤176 ± 31) compared to UC and TT treatments across all evaluation phases and sample days (Figure 3).

### 3.2. Aphid Abundance over Time in the 2022 Season

**Figure 4:**
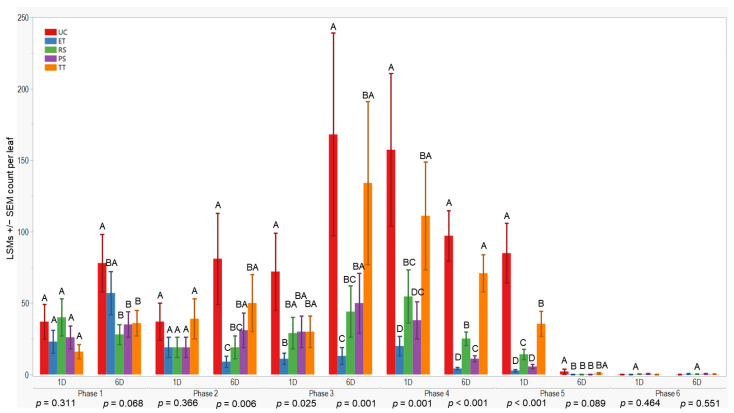
LSMs with SE counts per leaf of wingless in the 2022 season. LSMs with the same letter are not significantly different from each other (*p* > 0.05).

All treatments initially displayed similar results on the first and sixth days after application in the first evaluation phase (*p* = 0.311; 0.068). However, variances in wingless aphid counts emerged by the sixth day after application in the second evaluation phase (*p* = 0.006). The UC treatment exhibited higher counts (81 ± 32) compared to ET. Throughout the third evaluation phase, ET consistently maintained lower counts compared to RS, while UC and TT showed higher counts (≤168 ± 71). These trends persisted through subsequent evaluation phases. To summarize, ET plots consistently recorded lower aphid counts, followed by PS and RS treatments (≤57 ± 15), whereas UC and TT treatments consistently displayed higher counts overall (Figure 4).

### 3.3. Cumulative Aphid Days

**Figure 5:**
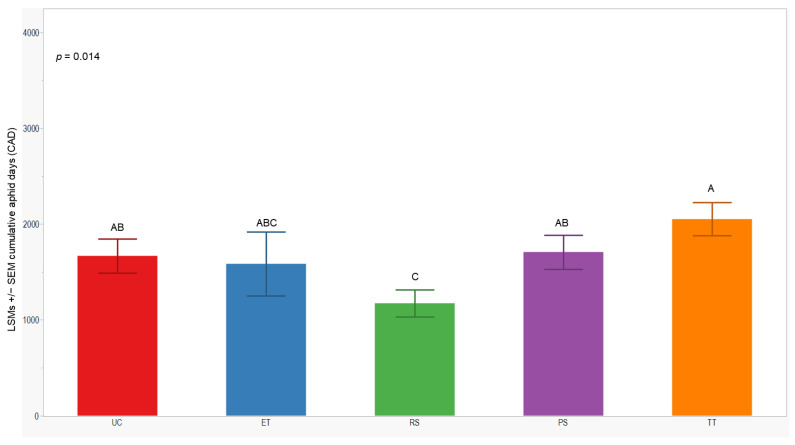
LSMs with SE of CADs: wingless in the 2021 season. LSMs with the same letter are not significantly different from each other (*p* > 0.05).

**Figure 6:**
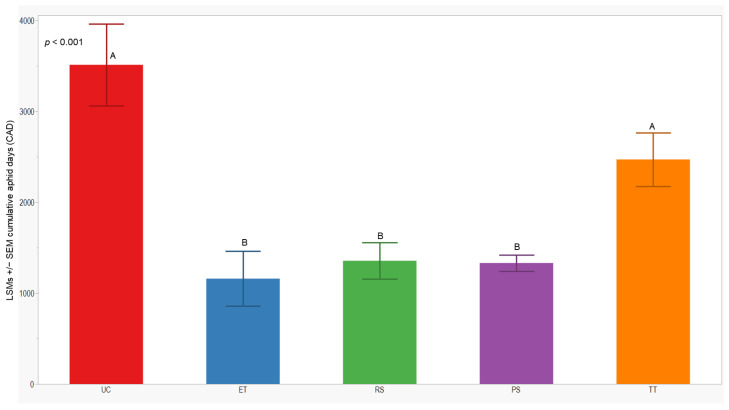
LSMs with SE of CADs: wingless in the 2022 season. LSMs with the same letter are not significantly different from each other (*p* > 0.05).

We observed significant differences in cumulative aphid days (CADs) across both years (*p* = 0.014; <0.001). In 2021, the separation of least-squares means (LSMs) for CADs indicated that the number of plots managed with the RS treatment was significantly lower (1173 ± 142) than those managed with the UC, PS, and TT treatments. In 2022, the CADs in the ET, RS, and PS treatments was significantly lower (≤1357 ± 200) compared to the UC and TT treatments (Figure 5 and Figure 6).

### 3.4. Cumulative Sprayed Plants and Grain Yield

**Figure 7:**
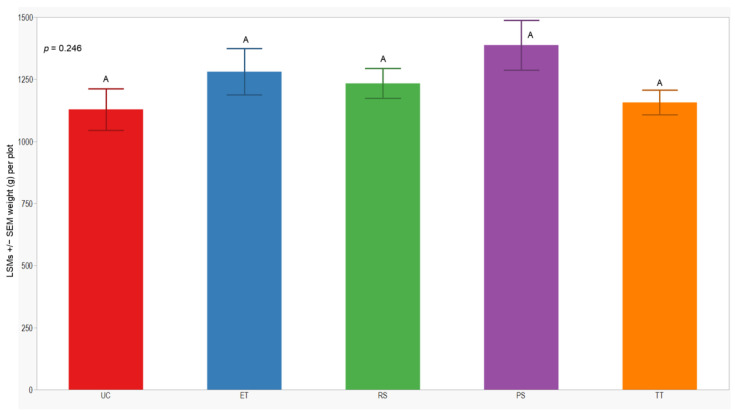
LSMs with SEs of yield in the 2021 season. LSMs with the same letter are not significantly different from each other (*p* > 0.05).

**Figure 8:**
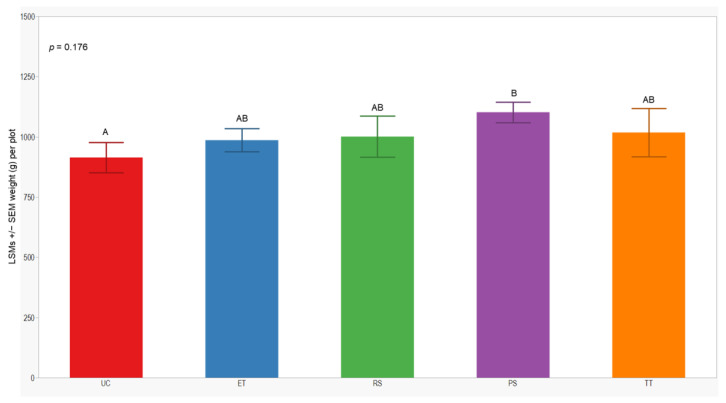
LSMs with SE of yield in the 2022 season. LSMs with the same letter are not significantly different from each other (*p* > 0.05).

In both seasons, we observed a higher number of cumulative sprayed plants in plots treated with ET (2588 plants) compared to those treated with RS (612 plants), PS (612 plants), and TT (214 plants). Notably, the number of cumulative sprayed plants in plots treated with RS, PS, and TT was sprayed 4 times lower than in the plots treated with ET in both seasons (Table 3). Regarding the yield response variable, measured by the dry weight of the grain standardized to 14% humidity, no significant differences were observed between treatments in either year of this study (*p* = 0.246; 0.176). However, the lowest yield was consistently observed in the UC treatment (1129 ± 84; 914 ± 62) in both years of this study (Figure 7 and Figure 8).

## 4. Discussion

In this study, we demonstrated a reduction in the number of wingless aphids in plots where most plants were sprayed based on the established economic threshold (≥40 mean sorghum aphids per leaf), as well as in the RS and PS management strategies, where only individual plants were sprayed. Despite not observing aphid densities reaching an economic threshold level in 2020 and moderate numbers during the 2021 and 2022 growing seasons (i.e., CADs less than 5000), our results show that aphid suppression varied among sprayed treatments. 

Overall, lower wingless counts were observed across the evaluation phases and sample days in plots managed with ET, followed by those managed with the RS and PS treatments. However, the number of cumulative sprayed plants found in plots managed with RS and PS was sprayed 4 times less than in plots managed with ET (Table 3). This suggests that compared with standard whole-plot applications based on an established economic thresholds (ET), spraying individual plants based on RS or PS management can reduce aphids and potentially mitigate the environmental effects of pesticides [14]. However, these results are based on plot studies, which further need to be evaluated at the field level.

Furthermore, we observed a considerable variation in wingless aphid density between the evaluation phases in both seasons (Figure 3 and Figure 4). This suggests that the population dynamics of sorghum aphids changed over time [14]. According to Lytle et al. [23], interannual fluctuations in aphid populations are characteristic of many field crops where aphid populations are critical pests. For instance, economic infestations of sorghum aphids in Kansas have been less frequent and severe since 2017 [24]. The combination of different management practices, including host–plant resistance, planting date, and the adaptation of aphids’ natural enemies, influences their reproduction and propagation in sorghum fields [17,24,25]. We consider the planting date to be a factor that influenced the aphid pressure at our field sites because early planting (May–June), similar to our planting dates, helps to reduce aphid infestations in Kansas [15]. Therefore, this can be attributed to our study’s low aphid wingless pressure and CADs.

In both years of this study, the calculated CADs were lower in all the treated plots than in the UC treatment plots; however, the CADs in the UC treatment plots comprised less than 5000 CADs, resulting in no yield differences. This suggests that our pest pressure was not high enough to observe differences in yield. Similar studies evaluating the effect of insecticides and cultural management strategies on sorghum aphids revealed yield differences when the CADs were above 10,000 [23,26]. However, it also shows that we cannot affect yield when we manually infest aphid wingless, suggesting that the timing of infestation was too late into sorghum development, which is less susceptible to aphid injury than vegetative growth stages [19].

There is a need to identify alternatives for managing sorghum aphids to reduce further insecticide resistance and environmental effects [14,23]. Our current study provides information about the development of alternative management strategies for sorghum aphids. The RS and PS treatments based on individual plant applications could be alternatives for aphid management when the infestation intensity and pressure can injure sorghum plants. Developing and deploying these strategies will further improve the management options for this migratory and invasive pest.

## 5. Conclusions

Treating the entire sorghum field with insecticides could increase the risk of pest resistance and reduce the number of natural enemies contributing to aphid natural predation. Individual plant applications could be an alternative to common management strategies (i.e., treating the entire field) to reduce sprayed plants and improve aphid management. Our suggested approach revealed that RS and PS treatments based on individual plant applications can reduce aphid wingless populations. These results can inform decision-making processes for growers that can balance pest control and non-target environmental effects. 

## Figures and Tables

**Table 1:** Abbreviations of the treatments along with their descriptions and the cumulative number of sprayed plants (CSP) over both years.

Treatment Abbreviation	Treatment Name	Treatment Description and CSP
UC	Untreated control	No insecticide applications were made. No plants were sprayed.
ET	Economic threshold	All plants in the two middle rows of the plot were sprayed based on an economic threshold of ≥40 mean sorghum aphid wingless/leaf on 12 randomly evaluated plants. A CSP of 2588 was sprayed.
RS	Randomly sprayed	Consisted of the same number of plants treated as in PS but randomly sprayed plants regardless of sorghum aphid wingless presence. A CSP of 612 was sprayed.
PS	Plant-specific	Consisted of specific plants sprayed upon visually confirming the presence of wingless (≥1 sorghum aphid wingless/leaf) on 12 randomly evaluated plants. A CSP of 612 was sprayed.
TT	Tally threshold	Consisted of individual plants sprayed based on an economic threshold per plant basis of ≥40 sorghum aphid wingless/plant on 12 randomly evaluated plants. A CSP of 214 was sprayed.

**Table 2:** *p*-value for the type III test of the fixed effect of wingless aphid counts by season.

*p*-Value for Type III Test of Fixed Effect	2021 Season Wingless Aphids	2022 Season Wingless Aphids
Evaluation baseline	0.492	0.285
Block	0.018	0.452
Treatment (Trt)	0.068	<0.001
Evaluation phase (EP)	<0.001	<0.001
Sample day (D)	<0.001	<0.001
Trt × EP	<0.001	<0.001
Trt × D	0.098	0.113
EP × D	<0.001	<0.001
Trt × EP × D	<0.001	0.288

**Table 3:** Cumulative number of sprayed plants (CSP) in the 2021 and 2022 seasons by treatment.

Treatment Abbreviation	Treatment Name	2021 Season CSP	2022 Season CSP
UC	Untreated control	-	-
ET	Economic threshold	1388	1200
RS	Randomly sprayed	280	332
PS	Plant-specific	280	332
TT	Tally threshold	92	122

## Data Availability

The collected dataset used in this study will be available upon request from the main author.
